# Psychiatric manifestations of measles encephalitis: About a case report

**DOI:** 10.1192/j.eurpsy.2022.2270

**Published:** 2022-09-01

**Authors:** M. Bouhamed, I. Feki, A. Guermazi, F. Guermazi, R. Sallemi, J. Masmoudi

**Affiliations:** Hospital university of HEDI CHAKER, Psychiatry A Department, Sfax, Tunisia

**Keywords:** Psychiatric manifestations, case report, measles encephalitis

## Abstract

**Introduction:**

Acute measles encephalitis is a pathology of the central nervous system. It is most frequent in children but can also be described in adults. Given the rarity of this pathology, we present the case of this patient.

**Objectives:**

present a rare neuropsychiatric complication of measles

**Methods:**

Présentation d’un cas clinique d’encéphalite rougeoleuse et revue de la littérature

**Results:**

Mrs. HJ, 45 years old, without any somatic history, was followed for an antisocial personality with a substance use disorder. She consulted the emergency for psychomotor agitation, a fever of 39, and a rash on the face, thorax, and limbs. At the psychiatric interview, she was disoriented and very unstable. She seemed to be hallucinating.

The brain imaging and the lumbar puncture (CT scan and brain MRI) were without abnormality. The rapid test (HIV) was negative and the biological check-up showed a hyperleukocytosis at 12660 and a crp at 138. The patient was put on double antibiotic therapy.

The evolution was marked by the non-improvement of the symptomatology with the persistence of agitation. Her speech was almost absent with a refusal to answer and to execute orders. She maintained certain postures. The patient was put on 400 mg of amisulpride.

After recovery of the viral serology, the diagnosis of a measles encephalopathy was confirmed (IgM positive) and the patient improved after a few days of hospitalization and was addressed to the psychiatric outpatient clinic.

**Conclusions:**

Measles encephalitis is a rare but serious complication that requires multidisciplinary management

**Disclosure:**

No significant relationships.

